# Aspirin in Primary Prevention of Cardiovascular Disease and Cancer: A Systematic Review of the Balance of Evidence from Reviews of Randomized Trials

**DOI:** 10.1371/journal.pone.0081970

**Published:** 2013-12-05

**Authors:** Paul Sutcliffe, Martin Connock, Tara Gurung, Karoline Freeman, Samantha Johnson, Kandala Ngianga-Bakwin, Amy Grove, Binu Gurung, Sarah Morrow, Saverio Stranges, Aileen Clarke

**Affiliations:** 1 Warwick Medical School, University of Warwick, Coventry, Warwickshire, England; 2 Oxford Medical School, University of Oxford, Oxford, England; University of British Columbia, Canada

## Abstract

**Background:**

Aspirin has been recommended for primary prevention of cardiovascular disease (CVD) and cancer, but overall benefits are unclear. We aimed to use novel methods to re-evaluate the balance of benefits and harms of aspirin using evidence from randomised controlled trials, systematic reviews and meta-analyses.

**Methods and Findings:**

Data sources included ten electronic bibliographic databases, contact with experts, and scrutiny of reference lists of included studies. Searches were undertaken in September 2012 and restricted to publications since 2008. Of 2,572 potentially relevant papers 27 met the inclusion criteria. Meta-analysis of control arms to estimate event rates, modelling of all-cause mortality and L'Abbé plots to estimate heterogeneity were undertaken. Absolute benefits and harms were low: 60-84 major CVD events and 34-36 colorectal cancer deaths per 100,000 person-years were averted, whereas 46-49 major bleeds and 68-117 gastrointestinal bleeds were incurred. Reductions in all-cause mortality were minor and uncertain (Hazard Ratio 0.96; 95% CI: 0.90-1.02 at 20 years, Relative Risk [RR] 0.94, 95% CI: 0.88-1.00 at 8 years); there was a non-significant change in total CVD (RR 0.85, 95% CI: 0.69-1.06) and change in total cancer mortality ranged from 0.76 (95% CI: 0.66-0.88) to 0.93 (95% CI: 0.84-1.03) depending on follow-up time and studies included. Risks were increased by 37% for gastrointestinal bleeds (RR 1.37, 95% CI: 1.15-1.62), 54%-66% for major bleeds (Rate Ratio from IPD analysis 1.54, 95% CI: 1.30-1.82, and RR 1.62, 95% CI: 1.31-2.00), and 32%-38% for haemorrhagic stroke (Rate Ratio from IPD analysis 1.32; 95% CI: 1.00-1.74; RR 1.38; 95% CI: 1.01-1.82).

**Conclusions:**

Findings indicate small absolute effects of aspirin relative to the burden of these diseases. When aspirin is used for primary prevention of CVD the absolute harms exceed the benefits. Estimates of cancer benefit rely on selective retrospective re-analysis of RCTs and more information is needed.

## Introduction

Cancer and cardiovascular disease (CVD) are a heavy burden worldwide in morbidity, mortality and cost [1]. Use of primary prevention measures therefore have the potential for a large impact. A number of randomised controlled trials (RCTs) and guidelines have been published on use of aspirin for primary CVD prevention [2,3,4,5,6,7,8,9,10,11]. More recently attention has also focused on the possibility that prophylactic aspirin may have a role in the primary prevention of cancer, especially colorectal cancer although the mechanisms underlying a potential chemo-preventive effect are unclear [12,13]. 

Unwanted or harmful effects such as bleeding and stomach pain can also result from taking aspirin [14]. It is particularly important to know the risk of harmful effects when considering an intervention for primary prevention since by definition it will be used by a population people who are well and free from CVD or cancer.

Although internationally guidelines have adopted differing stances on prophylactic aspirin, no guidelines currently recommend routine use of aspirin across the adult population for primary prevention for either cancer or CVD. American Heart Association guidelines recommend aspirin for patients at ‘high risk’ of cardiovascular events (those with a 10-year risk of 6-10%) [15]. 

With regard to cancer prevention, the US National Cancer Institute states that research is ongoing to determine the role of aspirin in the prevention of cancer [16], and the US Preventive Services Task Force (USPSTF) recommends against the routine use of aspirin and non-steroidal anti-inflammatory drugs (NSAIDs) to prevent colorectal cancer in individuals at average risk [17].

Previous systematic reviews have either addressed aspirin for primary prevention of CVD or more recently have focused on aspirin in primary prevention of cancer. No overview has synthesized evidence from both sets of reviews and meta-analyses. We aimed to fill this gap and to extend previous analyses in this area using alternative methods. We undertook – a) meta-analysis of control arms so as to use pooled estimates in the calculation of event rate differences between aspirin and control groups; b) modeling of the impact of aspirin on life time all-cause mortality; and c) L’Abbé plots to explore between-study heterogeneity. 

## Methods

Standard systematic review methodology was used. This systematic review was guided by a protocol that was prepared a priori and externally reviewed prior to use.

### Data Sources and Searches

We searched electronic bibliographic databases, contacted experts in the field, and scrutinised references of included studies. An iterative procedure was used to develop the search strategy covering the concepts 'aspirin' and 'prevention’ (see Table S1) with input from clinical advisors, an experienced information specialist and previous systematic reviews [18,20]. 

Searches, undertaken in September 2012 were performed in MEDLINE; MEDLINE In-Process & Other Non-Indexed Citations; EMBASE; Cochrane Database of Systematic Reviews (CDRS); CENTRAL; DARE, NHS EED, HTA databases (NHS-CRD); Science Citation Index and Conference Proceedings (Web of Science); UKCRN Portfolio Database and Clinical Trials.gov. Citation searches of included studies were undertaken using the Web of Science citation search. Reference lists of relevant studies and relevant review articles excluded at abstract were checked. Searches were restricted to RCTs, meta-analyses and systematic reviews since 2008, based on timing of the most recent comprehensive systematic reviews. 

### Study Selection

Titles and abstracts were assessed for inclusion by two reviewers independently with disagreements resolved by full publication review, consensus agreement and discussion with a third reviewer. RCTs, systematic reviews and meta-analyses of RCTs were included. Studies were defined as primary prevention if participants with previous CVD or relevant cancers were excluded (or were separately identifiable and could be excluded) or represented <20% of included participants. To be included, systematic reviews had to report data from studies separately and a minimum of 50% of studies had to be eligible RCTs. Systematic reviews had to report at least one of the following: a) search strategy; b) inclusion/exclusion criteria; c) method of quality assessment; or d) method of data synthesis. 

#### Population

Adults aged over 18 years without clinical CVD (established or symptomatic), or adults aged over 18 years without cancer (established or symptomatic).

#### Intervention

The intervention was aspirin (any dosage including alternate day therapy) taken prophylactically for primary prevention of cancer or CVD. Studies reporting aspirin combination therapy (e.g., aspirin combined with a second antithrombotic agent) were only included if separate placebo and aspirin-only treatment groups were reported separately; in which case only data from these groups were included. The comparator was placebo; no aspirin; no other treatment or normal care.

#### Outcomes

Outcomes of interest were: all-cause mortality; incidence and mortality of cardiovascular disease or cancer and any reported harms. 

### Data Extraction and Quality Assessment

Data were extracted independently by one reviewer and checked by a second reviewer (using an adapted extraction sheet [21] and information from previous reviews [18,19,20]). Summary tables listing all outcomes were constructed. Quality criteria were applied independently by two reviewers and an agreed overall quality assessment was determined for each paper. Systematic reviews were quality assessed using the NHS CRD tool [21] and RCTs were quality assessed using the Cochrane Risk of Bias tool [22]. 

### Data Synthesis and Analysis

A narrative overview and analysis of included systematic reviews and meta-analyses was undertaken, supplemented with further meta-analysis. Particular attention was focused on reporting of harmful events including overall numbers and proportions; the range of harmful events, definitions employed in primary studies. We found that the definition and nomenclature of various grades of bleeding varied somewhat between included systematic reviews; however there appeared broad similarity across systematic reviews and we have been used nomenclature adopted by review authors. Within the primary studies the ascertainment of bleeding was generally from patient questionnaires or from general practitioners’ records or was unclear. Several primary studies provided detail regarding bleeds; for example in the Women’s Health Study [9] data for GI bleeds requiring transfusion was reported and in the AAA study [10] a statistically significant increase in major bleeds in the aspirin group while there was no prospect of a significant difference developing for the primary outcome resulted in premature discontinuation of the trial.

Meta-analyses, including cumulative meta-analysis of studies to identify changes through time, study level meta-analyses to investigate relative influence of individual RCTs. Exploratory multi-variable meta-regression were undertaken. Analyses was undertaken using STATA version 11 software [23]. Because of clinical heterogeneity, a random effects model was used [24]. We meta-analysed risk of events in comparator arms of trials using fixed and random effects meta-analysis and used resulting pooled estimates to calculate event rate differences between arms (see below). We modelled the impact of aspirin on life time all-cause mortality and investigated heterogeneity amongst studies and the risk of events in each trial arm of using L’Abbé plots. Statistical heterogeneity beyond that expected by chance was estimated with I^2^ [25].

#### Quantifying absolute benefits and harms

We re-analysed reported study-level data (see Table S2 for methods used), so as to estimate effects of aspirin on the number of outcome events, taking into account years of follow-up. In the aggregated method we summed events and patients across studies in each trial arm (events per person) and divided this by estimated total follow up (each study follow up was weighted according to number of participants). In an alternative procedure we used the pooled estimate of risk of an event in the control arm (see Figure S1) together with the reported risk ratio or odds ratio for the outcome, to generate the difference in number of events. This was then adjusted to events per year by dividing by the weighted follow up. The two methods generally produced very similar results. Numbers needed to treat (NNT) and numbers needed to harm (NNH) were estimated [22]. Absolute differences in event rates were normalised to events averted or events incurred for 10,000 people followed up for 10 years and these were estimated for each outcome (all-cause mortality; cancer mortality; colorectal cancer mortality; myocardial infarction, stroke or cardiovascular mortality; total coronary heart disease; non-trivial bleed; major bleed; gastro-intestinal bleed; and haemorrhagic stroke). UK Office of National Statistics data26 were used to estimate of survival of a 50 year- old cohort. This was fitted with a Gompertz distribution. Estimates of the impact of aspirin on mortality were made by adjusting the Gompertz scale parameter according to hazard and odds ratios for all-cause mortality reported in the included studies.

## Results

**Figure 1:**
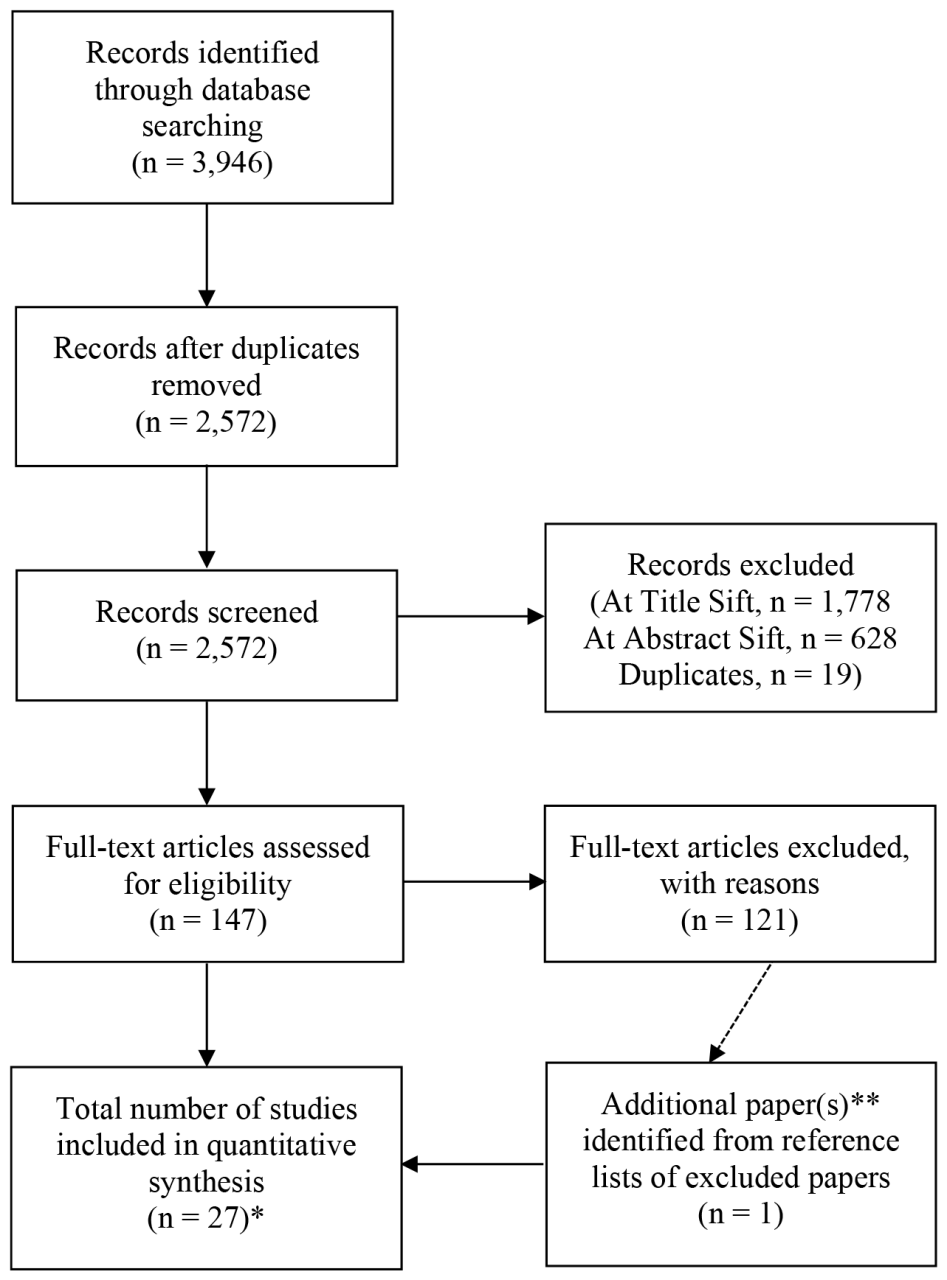
PRISMA flow diagram. *Key*: CVD=Cardiovascular diseases; RCT=Randomised controlled trial; SR=Systematic Review. *Of the 27 included publications: a) CVD, SR=9, RCT=3; b) Cancer, SR=6; and c) Diabetes, SR=7, RCT=2. **One paper was identified from assessment of reference lists of excluded papers, this had been excluded at abstract sift but was not considered relevant until reading the paper in full.

We identified 2,572 potentially relevant papers, of which 2,545 were removed at title, abstract, or full paper sift resulting in 27 papers which met the inclusion criteria (See Figure 1). These studies included 22 systematic reviews and meta-analyses of the use of aspirin for primary prevention of CVD (n = 9) or cancer (n = 6) and or CVD in patients with diabetes (n = 7). We looked for post 2008 RCTs in case our included systematic reviews had failed to include contemporaneously published primary studies. Five post 2008 RCTs were identified: three concerned use of aspirin for primary prevention of CVD, one of these was a constituent study in several of our included systematic reviews while the other two added no new data (one was a pilot study with limited outcome reportage and the other a post hoc modelling study); the other two post 2008 RCTs concerned aspirin for primary prevention of CVD in patients with diabetes and both were constituent studies in several of the our included systematic reviews. We found no post 2008 RCTs addressing primary prevention of cancer with aspirin and there were no pre 2008 RCTs where aspirin was the intervention for primary prevention of cancer. All identified cancer prevention systematic reviews assessed reduction in cancer incidence and mortality retrospectively through re-analysis of RCTs of aspirin for primary prevention of CVD.

### Characteristics of Included Studies

The nine systematic reviews and three post 2008 RCTs that assessed effects of aspirin on CVD mortality and incidence covered publication dates ranging from 2008 to 2012 (Table S8). All systematic reviews provided a clear aim; reporting of methods varied, with a number of studies not reporting: a) the search strategy (n = 3), b) inclusion criteria (n = 3); and c) quality assessment (n = 5). A broad range of outcome measures was reported. The majority of systematic reviews did not clearly distinguish between primary and secondary outcomes and there was a lack of clarity and consistency on definitions of harmful events (e.g., haemorrhagic stroke, gastrointestinal bleed, major bleed). These nine systematic reviews consistently reported on nine (or a sub-set of nine) RCTs depending on the year that meta-analysis was undertaken. The RCTs are: POPADAD [3], BDT [2], JPAD [4], AAA [10], HOT [5], TPT [6], PPP [7], PHS [8], and WHS [9], covering publication dates ranging from 1988 to 2010; further details of these nine primary studies are provided in Table 1.

**Table 1:** Aspirin dose and participant characteristics in the 9 RCTs of primary prevention.

**Study Year published**	Aspirin dose mg*	**Control arm**	**Gender% male**	**Design**	**Current smoker %**	**Additional therapies**	**Participants**
BDT 1988 [2]	300 or 500	No placebo	100	Open label	31	None	n = 5,139
PHS 1989 [8]	325 eod	Placebo	100	Double blind	11	Beta-carotene	n = 22,071
HOT 1998 [5]	75	Placebo	53	Double blind	16	Various**	n = 18,790
TPT 1998 [6]	75	Placebo	100	Double blind	41	Warfarin	n = 5,058
PPP 2001 [7]	100	No Placebo	42	Open label	15	Vitamin E	n = 4,495
WHS 2005 [9]	100 eod	Placebo	0	Double blind	10.1	Vitamin E	n = 39,876
						Beta-carotene	
POPADAD 2008 [3]	100	Placebo	44	Double blind	32	Antioxidant	n = 1,276
JPAD 2008 [4]	81 or 100	No placebo	55	Open label	4.4	None	n = 2,539
AAA 2010 [10]	100	Placebo	28	Double blind	33	None	n = 3,350

BDT=British Doctors Trial (BMJ 296,313); PHS=**P**hysician’s Health Study (NEJM 321, 129); HOT=Hypertension **O**ptimal **T**reatment (Lancet 351, 1755); TPT=Thrombosis Prevention Trial (Lancet 351, 233); PPP=Primary Prevention Project (Lancet 357, 89); WHS=**W**omen’s Health Study (NEJM 352, 1293); POPADAD=**P**revention **O**f **P**rogression of **A**rterial **D**isease And **D**iabetes (BMJ 337, a1840); JPAD=Japanese **P**rimary **P**revention of **A**therosclerosis with Aspirin for **D**iabetes (JAMA 300, 2134); AAA=Aspirin for **A**symptomatic **A**therosclerosis (JAMA 303, 841)

* aspirin taken each day unless specified; eod = every other day; ** therapies to achieve a target blood pressure

We identified six systematic reviews assessing the effect of aspirin on cancer mortality and incidence (Table S9). All these reviews used RCTs where the primary outcome was not cancer. Five of the six reviews were derived from the same team of investigators [13,20,27,28,29]. Quality was generally rated as high [13,20,28,29].

There were seven systematic reviews [30,31,32,33,34,35,36] and two post 2008 RCTs [3,4] assessing the effect of aspirin in the primary prevention of CVD events in patients with diabetes (Table S10). Both RCTs were constituent studies in most of the systematic reviews.

Details of the 22 systematic reviews can be found in tables S8, S9, and S10. A summary of quality assessment ratings in relation to study design and disease area is provided in Tables S3-S7. Quality ratings were in general high. 

### Evidence Synthesis

#### Relative effects: benefits

**Figure 2:**
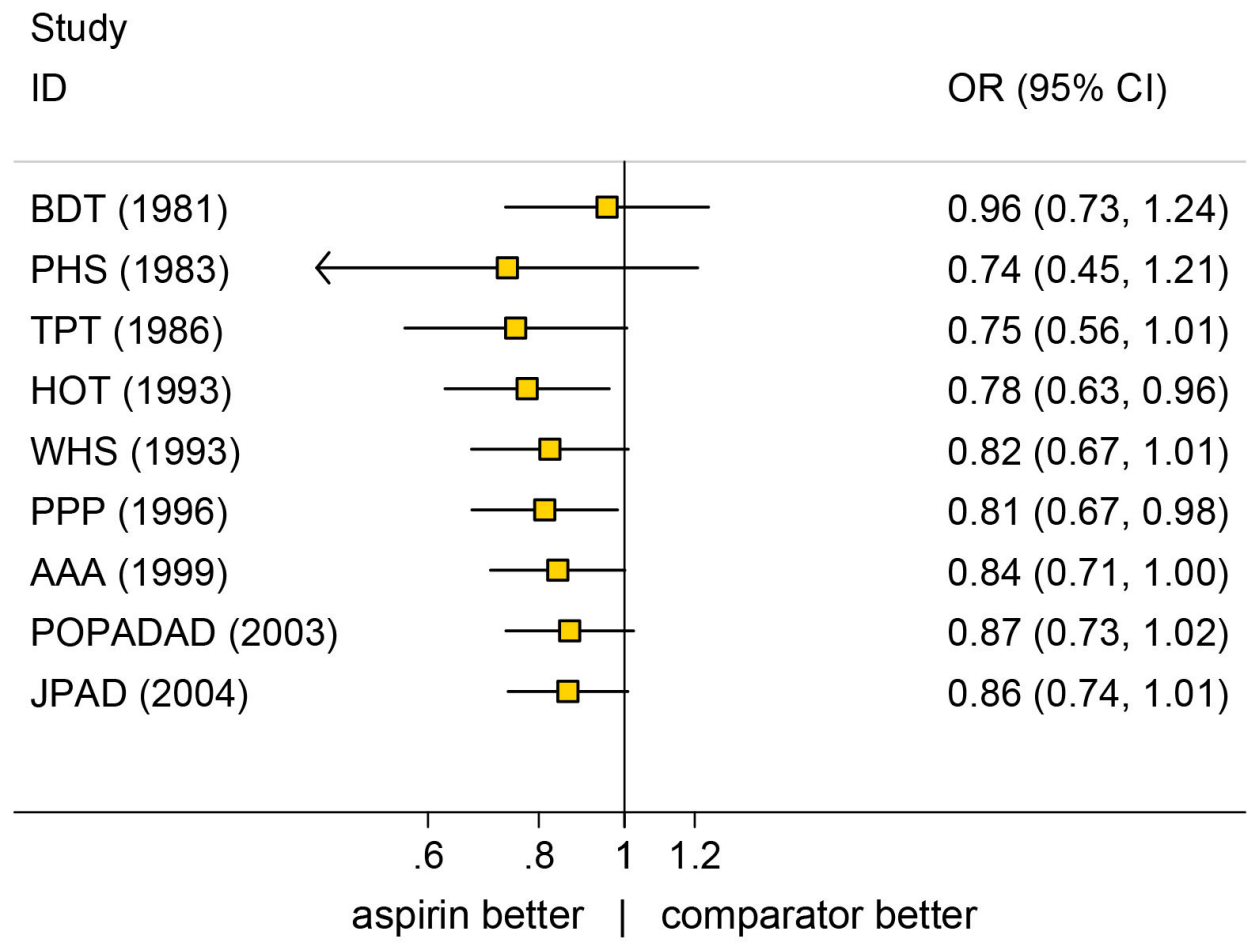
Cumulative random effects meta-analysis of odds ratio for total CHD. Studies arranged according to recruitment year (data from Seshasai et al., 2012) [38].

In CVD primary prevention, meta-analyses demonstrated reduced risks ranging from 6% for all-cause mortality, RR 0.94, 95% confidence interval (CI): 0.88 - 1.00 [37] to 10% for major cardiovascular events RR 0.90, 95% CI: 0.85 - 0.96 [19], while the odds ratio (OR) for total CVD included a null effect, or harm from aspirin (OR 0.85, 95% CI: 0.69 - 1.06 [18] and OR 0.86 95% CI: 0.74 - 1.01 [38])(See Table 2). In cumulative meta-analysis the odds ratio for total CVD appears gradually to have approached the null effect in recent years with accumulation of later studies (Figure 2). Early studies tended to be more favourable. This may be ascribed to improving treatments for CVD over the years or to changes in underlying risk and lifestyle factors as suggested e.g. by Seshasai et al. (2012) [38] and others. 

**Table 2:** Results from CVD and cancer systematic reviews: all comparisons aspirin vs. control.

	**Published studies and Reported pooled estimates**				**Re-analysis of reported data**	
**EVENT**	**Author (N studies)**	**Pooled estimate (95% CI)**	**NNTNNH**	**Absolute Difference (%/patient year)**	**Person years exposure for one less or one extra event**	**Events averted or events incurred for 10,000 persons followed up for 10 years**
All-cause mortality	Raju [37] (9)	RR 0.94 (0.88–1.00)	314**		2,752*	36*
					2,172**	46**
All-cause mortality	Berger [19] (9)	RR 0.94 (0.89–1.00)	318**		2,996*	33*
					2,198**	46 **
All-cause mortality	Rothwell [28] ^ (8)	OR 0.92 (0.85–1.00)				85*
						75**
All-cause mortality	Rothwell [28] ^^ (3)	HR 0.96 (0.90–1.02)				
Cancer mortality ~ 7 year follow up	Seshasai [38] (8)	OR 0.93 (0.84–1.03)	677**		5,974*	17*
					4,779**	21**
Cancer mortality	Rothwell [28] ^ (8)	OR 0.79 (0.68–0.92)				85*
						54**
Cancer mortality	Rothwell [28] ^^ (3)	HR 0.80 (0.72–0.88)				
Cancer mortality	Rothwell [20] ^ (51)	OR 0.84 (0.75–0.94)	319**			25* (36 assumes mean follow up 7 years)
						31** (44 assumes mean follow up 7 years)
Colo-rectal cancer death ~ 20 year follow up	Rothwell [13] (4)	OR 0.66 (0.51–0.85)		0.034***		34*36
				0.036		
MI / stroke / CV death	ATT [41] IPD (6)	RaR 0.88 (0.82–0.94)		-0.06	1,667	60
MI / stroke / CV death	Berger [19] (9)	RR 0.90 (0.85–0.96)	171**		1,676*	60*
					1,184**	84**
Total CHD	Seshasai [38] (9)	OR 0.86	226**		2,146*	47*
		(0.74–1.01)			1,564**	64**
Total CHD	Bartolucci [18] (9)	OR 0.85 (0.69–1.06)	NC		NC	NC
Non-trivial bleed	Seshasai [38] (9)	OR 1.31 (1.14–1.50)	146**		562	178*
					1010**	99**
Major bleed	Berger [19] (9)	RR 1.62	293**		2,082	48*
		(1.31–2.00)			2,208	49**
Major bleed	Raju [37] (7)	RR 1.66	312**		2078*	48*
		(1.41–1.95)			2186**	46**
Major bleed	ATT [41] IPD (6)	RaR 1.54 (1.30–1.82)		0.030	3333	30
GI bleed	Raju [37] (8)	RR 1.37 (1.15–1.62)	211*		853**	117**
					1476*	68*
Haemorrhagic stroke	Raju [37] (8)	RR 1.36 (1.01–1.82)	534*		10,516*	10*
					4,080**	25**
Haemorrhagic stroke	Berger [19] (8)	RR 1.35 (1.01–1.82)	1421*		11,165**	9**
					10,798*	9*
Haemorrhagic stroke	ATT [41] IPD (6)	RaR 1.32 (1.00–1.74)		0.01**^*#*^**		10**^*#*^**
				0.00818**^*##*^**		8**^*##*^**

CHD = coronary heart disease; MI = myocardial infarction; GI = gastrointestinal; RaR = rate ratio; RR = risk ratio; OR = odds ratio; HR = hazard ratio; NC = not calculated because individual patient and event numbers not reported.

* aggregate method; ** alternative method; *** aggregate data from Figure 1 of Rothwell 2010 [13] (119 colorectal deaths / 8282 aspirin users and 121colorectal deaths / 5751 aspirin “non-users”, over 20 years of follow up (including approximately 5 years of scheduled aspirin use); # based on rounded data; ## based on unrounded aggregate data; ^ assumes mean follow up of 10 years; ^^ follow up 20 years; Cancer mortality refers to death from any cancer.

Apparent cancer benefits appeared after about five years from start of treatment. The reported pooled OR for total cancer mortality was 0.93 (95% CI: 0.84 - 1.03) when mean follow up was at six to seven years [38]. With longer follow up (up to 20 years), a hazard ratio of 0.80, (95% CI: 0.72 - 0.88) has been reported [28]. The OR for within trial cancer death in eight trials (25,570 persons) was 0.79, 95% CI: 0.68 - 0.92 [28]. However, the large Women’s Health study and Physician’s Health study were omitted [39,40]. Together these studies reported on nearly 62,000 individuals and used alternate-day dosing [39], [ 40]. Relative beneficial effects were most striking for colorectal cancer mortality where an OR of 0.66 (95% CI 0.9-1.02) was reported [13] (see Table 2). This study also omitted the two largest studies where aspirin was given every other day [39], [ 40]. When these two large studies were included, estimates of colorectal cancer incidence decreased and became non-significant suggesting that aspirin might increase as well as reduce risk. The hazard ratio for all-cause mortality for three long-term studies at 20 years was also non-significant (0.96, 95% CI: 0.90 - 1.02) [28]. The authors hypothesised that this negative result might be due to a rebound effect subsequent to withdrawal from aspirin use.

L’Abbé plots indicated considerable heterogeneity between studies in event rates for all outcomes (all-cause mortality, cancer mortality, major CVD events) (see Figure S2). Meta-analyses in which each study in turn was omitted from pooled estimates indicated that several large studies (e.g., WHS [9], PHS [8]) were highly influential in determining results of meta-analyses for some outcomes (Figure S2).

#### Relative effects: harms

Study level meta-analyses of nine trials indicated a 62% RR 1.62, 95% CI 1.31 to 2.00) [19] and 66% increased risk of a major bleed from aspirin usage (RR 1.66, 95% CI 1.41 to 1.95) [37]. Individual patient data (IPD) meta-analysis of six trials suggested a similarly increased event rate of 54% (Rate Ratio 1.54, 95% CI 1.30 to 1.82) [41]. Increased risk of a gastrointestinal bleed was estimated to be 37% (study level analysis of 8 trials, RR 1.37, 95% CI 1.15 to 1.62) [37]. The estimated increased risk of a haemorrhagic stroke ranged from 32% (IPD analysis of 6 trials; Rate Ratio 1.32, 95% CI 1.00 to 1.74) [41] to 37% (study level analysis of 8 trials; RR 1.37, 95% CI 1.15 to 1.62) [37]. 

#### Absolute number of events averted or incurred through use of aspirin

The ATT authors [41] reported the rate of averted and of incurred events as % / person year; thus an absolute difference (aspirin – control) of -0.06% is equivalent to 0.06 events avoided per 100 patient years of exposure. However, this analysis included only six of the core nine trials currently available [41]. Based on our re-analysis using reported studies [13,19,20,28,37,38] we found the numbers of events averted after follow up of 10,000 people over ten years were: 33 to 46 deaths (any cause), 60 to 84 major cardiovascular events (MI or stroke or cardiovascular death), 47 to 64 total CVD events (major cardiovascular events as composite of non-fatal MI, non-fatal stroke, or cardiovascular death), 34 to 36 colorectal cancer deaths and 17 to 85 deaths from any cancer (the first estimate from study level data reported by Seshasai [38] with about 7 years mean follow up and the second from IPD analysis data reported by Rothwell [28] with about 10 years follow up). Cancer outcomes were mainly ascertained from retrospective analysis of medical records. The number of harmful events incurred per 10,000 people for 10 years were: 46 to 49 major bleeds, 68 to 117 gastrointestinal bleeds, and 8 - 10 haemorrhagic strokes. Estimated events (both positive and negative) occurred at the rate of a few tens of events per 100,000 person-years, other than gastrointestinal bleeds which appear to occur at somewhat higher rates of 68 to 117 per 100,000 person-years. These values represent “best point” estimates and although based on the most complete available systematic review evidence are associated with appreciable uncertainty. Table 2 lists these findings.

Composite primary outcomes in the primary prevention of CVD in diabetes show that for all seven of the included systematic reviews and meta-analyses, all upper 95% confidence intervals included the possibility of no improvement, and for some, confidence intervals clearly implied the possibility of a greater risk from aspirin [30,31,32].

**Figure 3:**
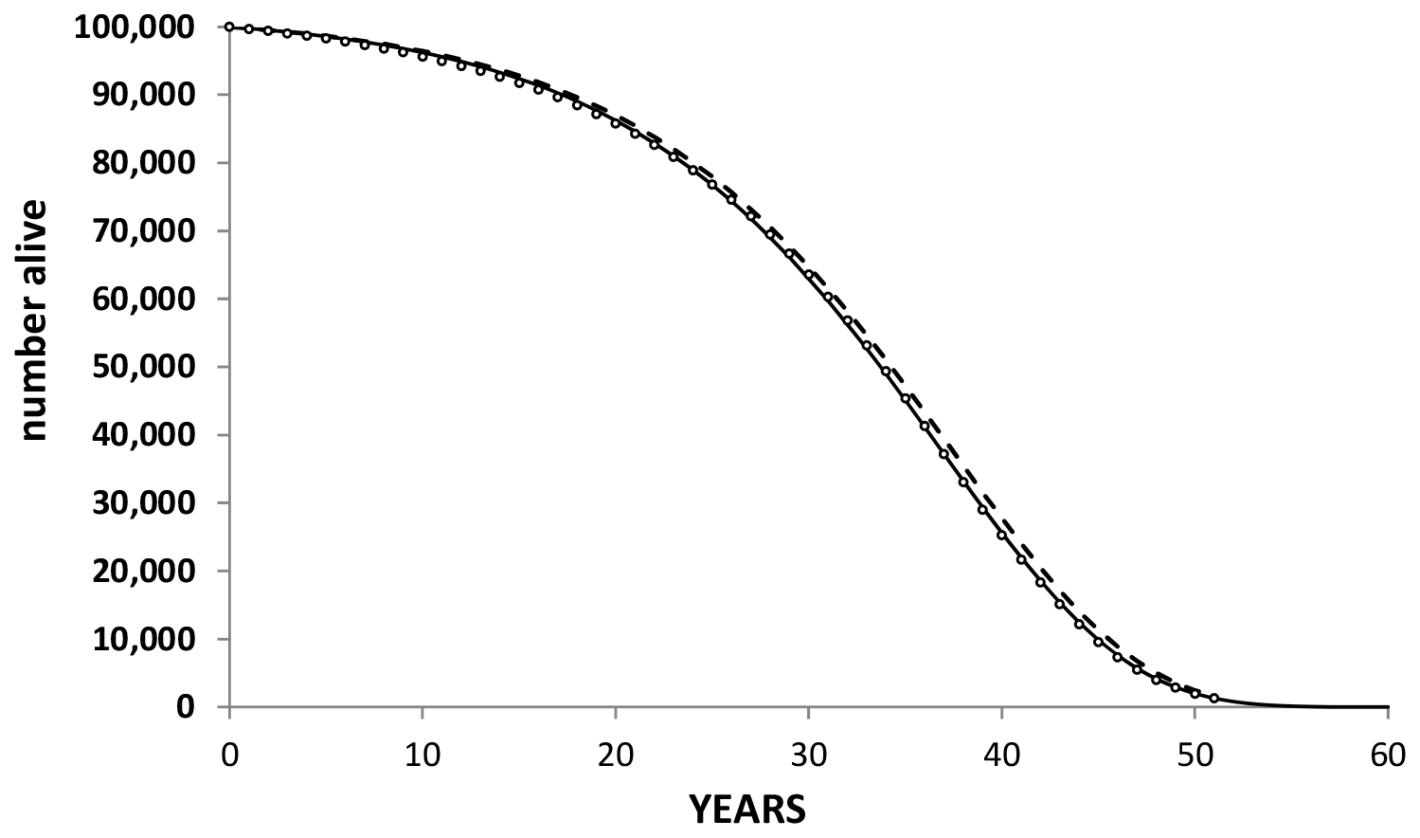
Gompertz fit to the ONS data. Symbols represent the UK ONS survival for 50 year old individuals; the solid line is a Gompertz fit to the ONS data; the dashed line represents the survival of aspirin users based on a ten year OR for all-cause mortality of 0.94 (Seshasai [38] & Berger [19]) and modelled keeping the scale parameter for the Gompertz fit constant. The difference in area under the solid and dashed curves represents the mean gain in life over a life time horizon.

We estimated mean life years gained over a life time horizon using all-cause mortality data from the reviewed studies (Table 2) and survival data [26] for 50-year-olds described using a Gompertz distribution (Figure 3). Using the reported pooled odds ratio of 0.94 for all-cause mortality from previous meta-analyses [19,38] and assuming an average of 10 years follow up, a mean life time gain of about 6 months is generated (difference in area under curves) (Figure 3). By applying 20 year all-cause mortality HR of 0.96 based on IPD [28] a lower gain of approximately 4.3 months is obtained. It should be born in mind that the upper 95% confidence interval on this hazard ratio encompasses a null effect. Thus this long term all-cause mortality data does not provide a compelling case for aspirin protection against CVD and cancer mortality. 

## Discussion

### Summary of main findings

We aimed to overview published systematic reviews that addressed the issues of primary prevention of cancer and CVD with aspirin and to supplement these with any evidence published subsequent to their publication. We investigated and synthesized evidence on the risks and benefits of aspirin for the primary prevention and found that benefits of aspirin for primary prevention of CVD are modest, remain statistically uncertain, and are an order of magnitude less than those observed for secondary prevention of CVD. In contrast, harms (especially bleeding) occur at a higher frequency (apparently very high frequency in some populations) and estimated rates are based on stronger evidence. 

Investigations which use a mix of IPD and study level analyses of RCTs now point to a possible primary protection against several cancers (notably colon cancer) emanating after about five years of aspirin use. However, these studies should be viewed with caution, since they excluded data from the two largest primary prevention trials [8,9], each of which show little evidence of cancer protection by aspirin after ≥ 10 years follow-up [39,40]. Because these are retrospective re-analyses of studies aimed at a different primary outcome and where rigorous case ascertainment after longer term follow-up cannot be verified, selection bias may be operating. That is, in practice people who suffer gastrointestinal problems or minor bleeding may self-select to discontinue aspirin use, disrupting the benefits of the equivalence conferred by randomisation between intervention and control groups. 

We found that absolute benefits and risks of aspirin use, estimated using various methodologies, are rare, (usually tens of events per 100,000 person-years of follow-up) compared to the total burden of the relevant diseases in the population and are finely balanced. Estimated values represent best estimates and although based on the most complete available systematic review evidence are associated with appreciable uncertainties. 

### Limitations in the evidence base

The published RCT evidence-base does not appear to have grown since the most recent completed trial [10]. This evidence has been subject to intense systematic review and meta-analysis including many study level meta-analytic investigations, a landmark IPD meta-analysis for CVD [41] and multiple publications by Rothwell and colleagues for cancer [13,20,28,29]. In general, the published meta-analyses appear to be well conducted and are up to date. However, inferences and conclusions differ from study to study. A recurring problem with the primary evidence base is the possibility of over the counter use of aspirin by patients in the control arms of the studies; there appears to be little evidence regarding the extent of this potential contamination.

### Strengths and limitations of this review

We undertook comprehensive searches and thorough systematic review methods following recognised guidelines. We evaluated all studies and re-analysed meta-analytic findings. We limited searches to 2008 or after, nevertheless because of the intense interest that this subject has generated and the cataloguing of all primary research in so many systematic reviews, we are confident that we have not omitted any major relevant randomised controlled trials or systematic reviews. A further limitation is our reliance on study level systematic reviews in which person years of follow up are not accurately ascertainable. However, estimates of number of events averted or incurred through aspirin use calculated from data in study level meta-analyses did not differ substantially from estimates based on IPD level meta-analyses, where person years of follow up were more accurate.

### Research needs

Clinical trials of primary prevention with aspirin have accumulated about two thirds of a million person years of observation and analysis suggestive of a considerable expenditure of resources. Several potentially relevant on-going trials are underway, with expected completion dates between September 2013 and June 2019 (e.g. ARRIVE [42], ASCEND [43], ASPREE [44], ACCEPT-D [45], CARING [46]; including large RCTs of the potential benefits of aspirin in the prevention of cancer. 

Avenues for future research include: (1) investigation of the impact of different dose regimens on cardiovascular and cancer outcomes; (2) further investigation in specific subgroups stratified according to reliable risk assessment tools; (3) expanding IPD meta-analysis of RCTs to the fullest extent by pooling data from variously publicly funded international investigations; and undertaking competing risks analysis (4) full cost effectiveness (utility) analysis with development of an economic model to quantify relative costs and benefits more fully. 

### Implications for practice

Many guidelines currently propose aspirin for prevention for those at high risk, but definitions of high risk vary [17,47,48,49]. At a population level, aspirin for primary prevention of CVD is associated with net harm due to increased potential for bleeding, while the results for benefits are not persuasive. For the primary prevention of cancer we consider that more information is needed.

## Conclusions

In the present review, after novel re-analyses, we have found that the benefit from regular aspirin use in primary prevention of CVD is modest while its use increases risk of haemorrhagic stroke and major and minor bleeding. Effects on cancer prevention have a long lead time and are at present reliant on retrospective re-analyses. New RCTs are underway which may clarify the extent of benefit of aspirin in reducing cancer incidence and mortality. 

## Supporting Information

Checklist S1 **PRISMA checklist.**
(DOC)

Figure S1 **Meta-analysis of risk of event in the control arms of studies used by authors of meta-analyses.**
(DOCX)

Figure S2 **Additional analyses and L’Abbe plots.** References S1. Additional references provided in Tables S8 and S9.
(DOCX)

Table S1 **Record of searches undertaken.**
(DOCX)

Table S2 **Quantifying absolute benefits and harms.**
(DOCX)

Table S3 **Summary table of quality assessment ratings of systematic reviews of aspirin for the primary prevention of CVD (n = 9).**
(DOCX)

Table S4 **Summary table of quality assessment ratings of RCTs of aspirin for the primary prevention of CVD (n = 3).**
(DOCX)

Table S5 **Summary table of quality assessment of systematic reviews of aspirin for the primary prevention of CVD in patients with diabetes (n = 7).**
(DOCX)

Table S6 **Summary table of quality assessment ratings of RCTs of aspirin for the primary prevention of CVD in patients with diabetes (n = 2).**
(DOCX)

Table S7 **Summary table of quality assessment ratings of systematic reviews of aspirin for the primary prevention of cancer (n = 6).**
(DOCX)

Table S8 **Summary characteristics of included CVD systematic reviews and RCTs.**
(DOCX)

Table S9 **Summary characteristics of included systematic reviews investigating aspirin in the primary prevention cancer.**
(DOCX)

Table S10 **Summary characteristics of included systematic reviews and RCTs investigating aspirin in the primary prevention cardiovascular events in patients with diabetes.**
(DOCX)

References S1 **Additional references provided in Tables S8 and S9.**
(DOCX)
