# Supplementary material for: Aspirin in Primary Prevention of Cardiovascular Disease and Cancer: A Systematic Review of the Balance of Evidence from Reviews of Randomized Trials
Source: PLoS One. 2013 Dec 5;8(12):e81970. doi: 10.1371/journal.pone.0081970 (PMC3855368; doi:10.1371/journal.pone.0081970)
Supplement: Figure S1 — Meta-analysis of risk of event in the control arms of studies used by authors of meta-analyses. (DOCX) [file pone.0081970.s002.docx]

**Figure S1. Meta-analysis of risk of event in the control arms of studies used by authors of meta-analyses**

Raju et al., 2011 [37] All cause mortality

Raju et al., 2011 [37] GI bleeds

Raju et al., 2011 [37] Haemorrhagic strokes

Raju et al., 2011 [37] Major bleeds

Berger et al., 2011 [19] Composite major cardiovascular event (MCE) end point ( included nonfatal MI, nonfatal stroke, or cardiovascular death)

Berger et al., 2011 [19] All cause mortality

Berger et al., 2011 [19] Major bleeds

Berger et al., 2011 [19] Haemorrhagic stroke

Seshasai et al., 2012 [38] Total CHD

Seshasai et al., 2012 [38] Non-trivial bleeds

Seshasai et al., 2012 [38] Cancer mortality

Seshasai et al., 2012 [38] Total bleeds (some double counting likely)

Rothwell et al., 2010 [13] Colorectal cancer mortality

Rothwell et al., 2011 [28] All cause mortality

Rothwell et al., 2011 [28] Cancer motality

Rothwell et al., 2012 [20] Cancer mortality
