# Supplementary material for: Aspirin in Primary Prevention of Cardiovascular Disease and Cancer: A Systematic Review of the Balance of Evidence from Reviews of Randomized Trials
Source: PLoS One. 2013 Dec 5;8(12):e81970. doi: 10.1371/journal.pone.0081970 (PMC3855368; doi:10.1371/journal.pone.0081970)
Supplement: Figure S2 — Additional analyses and L’Abbe plots. References S1. Additional references provided in Tables S8 and S9. (DOCX) [file pone.0081970.s003.docx]

**Figure S2. Additional analyses and L’Abbe plots**

1] Raju et al., 2011 [37] meta-analysis for all-cause mortality

2] Seshasai et al., 2012 [38] meta-analysis for cancer mortality

3] Berger et al., 2011 [19] meta-analysis for Cardio Vascular events

4] Seshasai et al., 2012 [38] meta-analysis for total Coronary Heart disease

5] Seshasai et al., 2012 [38] meta-analysis for total bleeds

6] Raju et al., 2011 [37] meta-analysis for haemorrhagic stroke

7] Raju et al., 2011 [37] meta-analysis for GI bleeds

8] Seshasai et al., 2012 [38] meta-analysis for non-trivial bleeds

B

Figures on the left show the influence on the estimated pooled effect size and 95% CIs of leaving out each named study from the meta-analysis. Figures on the right are L’Abbe plots indicating the heterogeneity of event rates in the studies included in the meta-analyses; dashed lines represent the pooled RR or OR estimate, the solid line represents a null effect size (RR or OR = 1).
