# Supplementary material for: Aspirin in Primary Prevention of Cardiovascular Disease and Cancer: A Systematic Review of the Balance of Evidence from Reviews of Randomized Trials
Source: PLoS One. 2013 Dec 5;8(12):e81970. doi: 10.1371/journal.pone.0081970 (PMC3855368; doi:10.1371/journal.pone.0081970)
Supplement: Table S1 — Record of searches undertaken. (DOCX) [file pone.0081970.s004.docx]

## Table S1. Record of searches undertaken

**Medline via OVID interface, searched on 19/09/2012**

| **Results** | **Search Type** | **Actions** |
| --- | --- | --- |
| 1 | exp *Aspirin/ | 19106 |
| 2 | (aspirin or acetylsalicyl* or "acetyl-salicyl*" or "acetyl salicyl*").tw. | 38918 |
| 3 | 1 or 2 | 41948 |
| 4 | (prevent* or prophyla*).tw. | 885027 |
| 5 | exp Primary Prevention/ | 105281 |
| 6 | 4 or 5 | 969687 |
| 7 | randomized controlled trial.pt. | 336449 |
| 8 | (random* or controlled trial* or clinical trial* or rct).tw. | 709686 |
| 9 | meta-analysis.pt. | 36189 |
| 10 | ("meta-analysis" or "meta analysis" or metaanalysis or "systematic review*").tw. | 60362 |
| 11 | 7 or 8 or 9 or 10 | 833781 |
| 12 | 3 and 6 and 11 | 2773 |
| 13 | limit 12 to (english language and humans) | 2397 |
| 14 | limit 13 to yr="2008 -Current" | 614 |

**Medline In Process via OVID interface, searched on 19/09/2012**

| **Results** | **Search Type** | **Actions** |
| --- | --- | --- |
| 1 | exp *Aspirin/ | 2 |
| 2 | (aspirin or acetylsalicyl* or "acetyl-salicyl*" or "acetyl salicyl*").tw. | 1732 |
| 3 | 1 or 2 | 1733 |
| 4 | (prevent* or prophyla*).tw. | 46556 |
| 5 | "primary prevent*".tw. | 606 |
| 6 | 4 or 5 | 46556 |
| 7 | randomized controlled trial.pt. | 449 |
| 8 | meta-analysis.pt. | 43 |
| 9 | (random* or "controlled trial*" or "clinical trial*" or rct).tw. | 49519 |
| 10 | (metaanalysis or "meta analy*" or "meta-analy*").tw. | 4026 |
| 11 | 7 or 8 or 9 or 10 | 51642 |
| 12 | 3 and 6 and 11 | 125 |
| 13 | limit 12 to english language | 116 |
| 14 | limit 13 to yr="2008 -Current" | 82 |

**Embase 1980 to 2011, Week 38 via OVID interface, searched on 19/09/2012**

**Results Search Type Action**

| 1 | exp acetylsalicylic acid/ | 137449 |
| --- | --- | --- |
| 2 | (aspirin or acetylsalicyl* or "acetyl-salicyl*" or "acetyl salicyl*").tw. | 87233 |
| 3 | 1 or 2 | 144909 |
| 4 | exp primary prevention/ | 22741 |
| 5 | (prevent* or prophyla*).tw. | 1117343 |
| 6 | 4 or 5 | 1124596 |
| 7 | randomized controlled trial/ | 329063 |
| 8 | ("random*or controlled trial*" or "clinical trial" or rct).tw. | 94960 |
| 9 | meta analysis/ | 65756 |
| 10 | (metaanalysis or "meta-analysis" or "meta analysis" or "systematic review*").tw. | 83512 |
| 11 | 7 or 8 or 9 or 10 | 490269 |
| 12 | 3 and 6 and 11 | 3852 |
| 13 | limit 12 to (human and english language) | 3338 |
| 14 | limit 13 to yr="2008 -Current" | 955 |

**Science Citation Index and Conference Proceedings via the Web of Science interface, searched on 19/09/2012.**

(aspirin or acetylsalicyl* or "acetyl-salicyl*" or "acetyl salicyl*") AND (prevent* or prophyla* or "primary prevent*") AND ("randomi?ed controll* trial*" or random* or "controlled trial*" or "clinical trial*" or rct or "systematic review*" or metaanalysis or "meta-analysis" or "meta analysis")

Refined by: Languages=( ENGLISH )

Timespan=2008-01-01 - 2012-09-19.

Databases=SCI-EXPANDED, CPCI-S.

Total retrieved: 1748

|  |
| --- |

**Database of Systematic Reviews and CENTRAL, searched via the Cochrane Library on 20/09/2012.**

1. aspirin or acetylsalicyl* or "acetyl salicyl*" or "acetyl-salicyl*":ti,ab,kw (Word variations have been searched)
2. prevent* or prophyla*
3. MeSH descriptor: [Aspirin] explode all trees
4. MeSH descriptor: [Primary Prevention] explode all trees
5. (#1 or #3) and (#2 or #4)
6. 2944 (not limited by date)

Reviews limited to 2008 onwards: 53

CENTRAL limited to 2008 onwards: 321

**DARE, NHS EED and HTA databases searched via the Centre for Reviews and Dissemination at** <http://www.crd.york.ac.uk/crdweb/SearchPage.asp> **on 20/09/2012**

(aspirin or acetylsalicyl* or "acetyl salicyl*" or "acetyl-salicyl*") and (prevent* or prophyla*)
Limited to 2008-

Results:

DARE: 128
HTA: 11
NHS EED: 34

Clinical Trials **(http://clinicaltrials.gov/)**

Clinical Trials database searched on 20/09/12 with no date restriction

(Aspirin AND primary) 797

United Kingdom Clinical Research Network's (UKCRN) Portfolio Database (<http://public.ukcrn.org.uk/search/>)

UKCRN searched using Title/Acronym field on 20/09/12 with no date restriction

Aspirin 27
