# Supplementary material for: Aspirin in Primary Prevention of Cardiovascular Disease and Cancer: A Systematic Review of the Balance of Evidence from Reviews of Randomized Trials
Source: PLoS One. 2013 Dec 5;8(12):e81970. doi: 10.1371/journal.pone.0081970 (PMC3855368; doi:10.1371/journal.pone.0081970)
Supplement: Table S2 — Quantifying absolute benefits and harms. (DOCX) [file pone.0081970.s005.docx]

**Table S2. Quantifying absolute benefits and harms**

The number of unwanted events (e.g. all-cause mortality) averted by taking aspirin, and the number of adverse events (e.g. bleeding) incurred from aspirin use, are best calculated using individual patient data taking into account the person-years of exposure to aspirin. However IPD is not available from study level meta-analyses. There are various ways of calculating the rate of averted or of incurred events from study level data. Here we have used two methods, described below, and have compared the results across systematic reviews according to outcome.

In the “aggregated” method the aggregated number of events (i.e. sum) across all included trials is divided by the aggregated number of persons. This is done separately for each arm (aspirin and control) to calculate “events / person” (E/p). The weighted average follow up time across all included trials (MFU) (for the intervention arm often equivalent to years of exposure) was calculated as:

MFU = ∑ [ MTFU * PT ] / ∑ PT

Where MTFU = mean follow up in each trial, and PT = total participants in each trial.

“Events / person year” (E/py) for each arm = [ E/p ] * [ 1/MFU ]. The difference between arms then generates the “events averted / person year of follow up or the extra events incurred / person year of follow up. Because these numbers are small we normalised the results to: (a) patients years exposure required for one less event or for one extra event, and (b) number of events averted or extra events incurred should 10,000 patients be followed up for 10 years.

For the “pooled” method we used the random effects pooled risk of event for the control arm (CR) determined using Meta Analyst version 3.1.3 Tufts Medical Centre: [*http://tuftscaes.org/meta_analyst/*](http://tuftscaes.org/meta_analyst/) (see following section).

If the systematic review reported pooled odds ratio (ORp) for the outcome then the calculation proceeds as:

Odds for an event in control arm = CO = CR/[1-CR]

Odds for an event in aspirin arm = AO = CO * ORp

Risk of an event in the aspirin arm = AR = AO / [AO + 1]

Difference in risk between arms = DR = AR – CR

Number needed to treat (or harm) = NNT(H) = 1/DR (i.e. 1 extra or less event requires NNT(H) persons to be treated with aspirin). Since this number requires MFU years of follow up then MFU * NNT(H) = “person years follow up for one less or one extra event”. Again because this number is small we normalised the results to: (a) “patient years follow up required for one less event” or for one extra event; and (b) “number of events averted or extra events incurred should 10,000 patients be followed up for 10 years”.

It has been suggested that the risk observed in the largest available trial may offer a suitable control risk estimate for the NNT calculations that are based on study level meta-analyses; in the face of considerable heterogeneity in control rates this method was not adopted here because the largest trial for many outcomes was the WHS which was atypical in having an alternate day dose regimen, a 100% female population and the longest follow up period.
