# Supplementary material for: Aspirin in Primary Prevention of Cardiovascular Disease and Cancer: A Systematic Review of the Balance of Evidence from Reviews of Randomized Trials
Source: PLoS One. 2013 Dec 5;8(12):e81970. doi: 10.1371/journal.pone.0081970 (PMC3855368; doi:10.1371/journal.pone.0081970)
Supplement: Table S3 — Summary table of quality assessment ratings of systematic reviews of aspirin for the primary prevention of CVD (n = 9). (DOCX) [file pone.0081970.s006.docx]

Table S3. Summary table of quality assessment ratings of systematic reviews of aspirin for the primary prevention of CVD (n = 9)

*Based on NHS Centre for Reviews and Dissemination (CRD)* [21]

| **Question** | **Adelman 2011 [50]** | **ATT 2009 [41]** | **Bartolucci 2011 [18]** | **Berger 2011 [19]** | **Raju 2011 [37]** | **Raju 2012 [51]** | **Selak 2010 [52]** | **Seshasai 2012 [38]** | **Wolff 2009 [11]** |
| --- | --- | --- | --- | --- | --- | --- | --- | --- | --- |
| 1. Are any inclusion/ exclusion criteria reported in the review? * *A minimum of ≥ 1 inclusion criterion and ≥ 1 exclusion criterion was required to score “Yes”* | Unclear^1^ | Yes | No | Yes | Yes | Yes | Unclear | Yes | Yes |
| 2. Is there evidence of a substantial effort to search for all relevant research? *A minimum of ≥ 1 search terms and ≥ 1 bibliographic database identified* | Yes | Unclear^2^ | No | Yes | Yes | Yes | No | Yes | Yes |
| 3. Is the quality of included studies adequately assessed? *Quality assessment tool was used (this could have been adapted from a standardised tool e.g. CASP, CRD, Cochrane, etc.)* | No | No | No | No | Yes | Yes | No | Yes | Yes |
| 4. Is sufficient detail of the individual studies presented? *All six listed baseline characteristics should be provided to score “Yes”* | Yes | Yes | Yes | Yes | Yes | Yes | No^6^ | Yes | Yes |
| *aspirin dose* | Yes | Yes | Yes | Yes | Yes | Yes | No | Yes | Yes |
| *aspirin frequency* | Yes | Yes | Yes | Yes | Yes | Yes | No | Yes | Yes |
| *number of participants* | Yes | Yes | Yes | Yes | Yes | Yes | No | Yes | Yes |
| *age* | Yes | Yes | Yes | Yes | Yes | Yes | No | Yes | Yes |
| *gender* | Yes | Yes | Yes | Yes | Yes | Yes | No | Yes | Yes |
| *length of follow-up* | Yes | Yes | Yes | Yes | Yes | Yes | No | Yes | Yes |
| 5. Are the primary studies summarised appropriately? *The two listed items should be provided to score “Yes”* | Yes | Unclear^3^ | Unclear^4^ | Yes | Yes | Yes | Yes | Yes | Yes^7^ |
| *the review primary outcome was presented* | Yes | Unclear^3^ | No^4^ | Yes | Yes | Yes | Yes | Yes | Yes |
| *quantitative results for the primary outcome were presented in sufficient detail* | Yes | Yes | No | Yes | Yes | Yes | Yes | Yes | Yes |
| 6. Was individual patient data analysed? | No | Yes | No | No | No | Yes^5^ | Yes^6^ | No | No |

^1^ No formal listing; criteria more or less implicit

^2^ The review stated “*Electronic searches established that no similar trials of aspirin had been reported since 2002*”

^3^ Many outcomes identified and analysed, a primary outcome not specified, review discussed the balance between benefits and harms each represented by various outcomes

^4^ The review stated: “*aspirin may have a differential effect on different aspects of cardiovascular (CV) disease”*; thus many outcomes were identified and analysed, a primary outcome not specified, the review discussed the balance between benefits and harms each represented by various outcomes

^5^ This paper was a review of other reviews and considered the IPD meta-analysis reported by the Antithrombotic Trialists in 2009

^6^ The study was based on a previous systematic review i.e., the IPD meta-analysis reported by the Antithrombotic Trialists in 2009 (see above)

^7^ Analytical framework and key questions were defined
