# Supplementary material for: Aspirin in Primary Prevention of Cardiovascular Disease and Cancer: A Systematic Review of the Balance of Evidence from Reviews of Randomized Trials
Source: PLoS One. 2013 Dec 5;8(12):e81970. doi: 10.1371/journal.pone.0081970 (PMC3855368; doi:10.1371/journal.pone.0081970)
Supplement: Table S4 — Summary table of quality assessment ratings of RCTs of aspirin for the primary prevention of CVD (n = 3). (DOCX) [file pone.0081970.s007.docx]

Table S4. Summary table of quality assessment ratings of RCTs of aspirin for the primary prevention of CVD (n = 3)

*Based on the Cochrane Risk of Bias tool* [22]

| **Question** | **Nelson 2008 [53]** | **Dorresteijin 2011 [54]** | **Fowkes 2010 [10]** |
| --- | --- | --- | --- |
| 1. Adequate sequence generation | Yes | Yes | Yes |
| 2. Adequate allocation concealment | Unclear | Yes | Yes |
| 3. Blinding (especially outcome assessment) | Yes (“*double blind*”) | Yes | Yes |
| 4. Incomplete outcome data addressed | Yes (*reported 12 month follow up attendance)* | Yes | Yes |
| 5. Free of selective reporting | Yes | Yes | Yes |
| 6. Free of other potential bias^1^ | Yes | Yes | Yes |

^1^ e.g. similarity at baseline, power assessment, conflict of interest
