# Supplementary material for: Aspirin in Primary Prevention of Cardiovascular Disease and Cancer: A Systematic Review of the Balance of Evidence from Reviews of Randomized Trials
Source: PLoS One. 2013 Dec 5;8(12):e81970. doi: 10.1371/journal.pone.0081970 (PMC3855368; doi:10.1371/journal.pone.0081970)
Supplement: Table S5 — Summary table of quality assessment of systematic reviews of aspirin for the primary prevention of CVD in patients with diabetes (n = 7). (DOCX) [file pone.0081970.s008.docx]

Table S5. Summary table of quality assessment of systematic reviews of aspirin for the primary prevention of CVD in patients with diabetes (n = 7)

*Based on NHS Centre for Reviews and Dissemination (CRD)* [*21*]

| **Question** | **Butalia et al. (2011) [33]** | **Calvin et al. (2009) [34]** | **De Berardis et al. (2009) [35]** | **Simpson et al. (2011) [36]** | **Stavrakis et al. (2011) [32]** | **Younis et al. (2010) [31]** | **Zhang et al. (2010) [30]** |
| --- | --- | --- | --- | --- | --- | --- | --- |
| 1. Are any inclusion/exclusion criteria reported in the review? *A minimum of ≥ 1 inclusion criterion and ≥ 1 exclusion criterion was required to score “Yes”* | Yes | Yes | Yes | Yes | Yes | Yes | Yes |
| 2. Is there evidence of a substantial effort to search for all relevant research? *A minimum of ≥ 1 search terms and ≥ 1 bibliographic database identified* | Yes | Yes | Yes | Yes | Yes | Yes | Yes |
| 3. Is the quality of included studies adequately assessed? *Quality assessment tool was used (this could have been adapted from a standardised tool e.g. CASP, CRD, Cochrane, etc.)* | Yes | Yes | Yes | Yes | Yes | Yes | No |
| 4. Is sufficient detail of the individual studies presented? *All six listed baseline characteristics should be provided to score “Yes”* | Yes | Yes | Yes | Yes | Yes | Yes | Yes |
| *aspirin dose* | Yes | Yes | Yes | Yes | Yes | Yes | Yes |
| *aspirin frequency* | Yes | Yes | Yes | Yes | Yes | Yes | Yes |
| *number of participants* | Yes | Yes | Yes | Yes | Yes | Yes | Yes |
| *age* | Yes | Yes | Yes | Yes | Yes | Yes | Yes |
| *gender* | Yes | Yes | Yes | Yes | Yes | Yes | Yes |
| *length of follow-up* | Yes | Yes | Yes | Yes | Yes | Yes | Yes |
| 5. Are the primary studies summarised appropriately? *The two listed items should be provided to score “Yes”* | Yes | Yes | Yes | Yes | Yes | Yes | Unclear |
| *the review primary outcome was presented* | Yes | Yes | Yes | Yes | Yes | Yes | No |
| *quantitative results for the primary outcome were presented in sufficient detail* | Yes | Yes | Yes | Yes | Yes | Yes | No |
| 6. Was individual patient data analysed? | No | No | No | No | No | No | No |
