# Supplementary material for: Aspirin in Primary Prevention of Cardiovascular Disease and Cancer: A Systematic Review of the Balance of Evidence from Reviews of Randomized Trials
Source: PLoS One. 2013 Dec 5;8(12):e81970. doi: 10.1371/journal.pone.0081970 (PMC3855368; doi:10.1371/journal.pone.0081970)
Supplement: Table S6 — Summary table of quality assessment ratings of RCTs of aspirin for the primary prevention of CVD in patients with diabetes (n = 2). (DOCX) [file pone.0081970.s009.docx]

Table S6. Summary table of quality assessment ratings of RCTs of aspirin for the primary prevention of CVD in patients with diabetes (n = 2)

*Based on the Cochrane Risk of Bias tool* [22]

| **Question** | **Belch et al. (2008) [3**] | **Ogawa et al. (2008) [4]** |
| --- | --- | --- |
| 1. Adequate sequence generation | Yes | Yes |
| 2. Adequate allocation concealment | Yes | Yes |
| 3.Blinding (especially outcome assessment) | Yes (*“double blind”)* | Open label study for patients; assessors blinded |
| 4. Incomplete outcome data addressed | Yes (*“All analyses were done on an intention to treat basis”*) | Yes (*“intention to treat principle”*) |
| 5. Free of selective reporting | Yes | Yes |
| 6. Free of other potential bias^1^ | Yes | Yes |

^1^e.g. similarity at baseline, power assessment, conflict of interest
