# Supplementary material for: Aspirin in Primary Prevention of Cardiovascular Disease and Cancer: A Systematic Review of the Balance of Evidence from Reviews of Randomized Trials
Source: PLoS One. 2013 Dec 5;8(12):e81970. doi: 10.1371/journal.pone.0081970 (PMC3855368; doi:10.1371/journal.pone.0081970)
Supplement: Table S7 — Summary table of quality assessment ratings of systematic reviews of aspirin for the primary prevention of cancer (n = 6). (DOCX) [file pone.0081970.s010.docx]

Table S7. Summary table of quality assessment ratings of systematic reviews of aspirin for the primary prevention of cancer (n = 6)

*Based on NHS Centre for Reviews and Dissemination (CRD)* [21]

| **Question** | **Mills et al. (2012) [55]** | **Algra et al. (2012) [27]** | **Rothwell et al. (2010) [13]** | **Rothwell et al. (2011) [28]** | **Rothwell et al. (2012) [29]** | **Rothwell et al. (2012) [20]** |
| --- | --- | --- | --- | --- | --- | --- |
| 1. Are any inclusion/exclusion criteria reported in the review? * *A minimum of ≥ 1 inclusion criterion and ≥ 1 exclusion criterion was required to score “Yes”* | Yes | Yes | Yes | Yes | Yes | Yes |
| 2. Is there evidence of a substantial effort to search for all relevant research? *A minimum of ≥ 1 search terms and ≥ 1 bibliographic database identified* | Yes | Yes | No^3^ | Yes | Yes | Yes |
| 3. Is the quality of included studies adequately assessed? *Quality assessment tool was used (this could have been adapted from a standardised tool e.g. CASP, CRD, Cochrane, etc.)* | No | Unclear^1^ | No^4^ | No^6^ | No^6^ | No^6^ |
| 4. Is sufficient detail of the individual studies presented? *All six listed baseline characteristics should be provided to score “Yes”* | Yes | Yes | Yes | Yes | Yes | Yes |
| *aspirin dose* | Yes | Yes | Yes | Yes | Yes | Yes |
| *aspirin frequency* | Yes | Yes | Yes | Yes | Yes | Yes |
| *number of participants* | Yes | Yes | Yes | Yes | Yes | Yes |
| *age* | Yes | Yes | Yes | Yes | Yes | Yes |
| *gender* | Yes | Yes | Yes | Yes | Yes | Yes |
| *length of follow-up* | Yes | Yes | Yes | Yes | Yes | Yes |
| 5. Are the primary studies summarised appropriately? *The two listed items should be provided to score “Yes”* | Yes | Yes^2^ | Yes^5^ | Yes^7^ | Yes^7^ | Yes^7^ |
| *the review primary outcome was presented* | Yes | Yes | Yes | Yes | Yes | Yes |
| *quantitative results for the primary outcome were presented in sufficient detail* | Yes | Yes | Yes | Yes | Yes | Yes |
| 6. Was individual patient data analysed? | No | Yes | Yes | Yes | Yes | Yes |

^1^ No formal assessment was attempted; however, methods used for ascertainment of cancers in each study were described in detail

^2^ Primary outcome implicit and abstract

^3^ Trials of aspirin versus control in the UK or Sweden in the 1980s and early 1990s were studied; however, how these were found or identified was not described

^4^ No formal assessment tool was used

^5^ Primary outcome implicit

^6^ No formal assessment was undertaken and no assessment tool was used.

^7^ Primary outcome implicit in title etc.
