# Supplementary material for: Aspirin in Primary Prevention of Cardiovascular Disease and Cancer: A Systematic Review of the Balance of Evidence from Reviews of Randomized Trials
Source: PLoS One. 2013 Dec 5;8(12):e81970. doi: 10.1371/journal.pone.0081970 (PMC3855368; doi:10.1371/journal.pone.0081970)
Supplement: Table S8 — Summary characteristics of included CVD systematic reviews and RCTs. (DOCX) [file pone.0081970.s011.docx]

**Table S8. Summary characteristics of included CVD systematic reviews and RCTs**

| **Systematic reviews (First author, year)** | **Aims** | **Methods** | **Outcomes** | **Adverse events*** |
| --- | --- | --- | --- | --- |
| Adelman, 2011 [50] | To examine gender differences in the primary prevention of stroke with aspirin | Search: Medline, guidelines from US, British and European organisations and citations from articles. Inclusion criteria: Not reported. Analysis: Study-level meta-analysis. Quality assessment: None | Combination of MI, stroke or vascular death; risk of haemorrhage was reported | Not clearly distinguished - haemorrhagic stroke |
| Baigent, 2009 [41] | To undertake an IPD analysis to compare primary with secondary intervention | Search: Electronic searches, not specified. Inclusion criteria: Trials on randomised comparison of aspirin versus no aspirin. Analysis: Collaborative meta-analysis of individual participant data. Quality assessment: None | Serious vascular event, defined as MI, stroke, or death from a vascular cause; major coronary event; any stroke; death from any cause; and major extracranial bleed; MI and strokes (fatal or nonfatal) | Not clearly distinguished - major extracranial bleed and haemorrhagic stroke |
| Bartolucci, 2011 [18] | To update previous six-trial meta-analysis with three most recent trials | Search: No systematic review methods, meta-analysis only. Inclusion criteria: Not reported . Analysis: Study-level meta-analysis. Quality assessment: None. | (1) total coronary heart disease (CHD) as nonfatal and fatal MI and death due to CHD; (2) nonfatal MI as confirmed MI that did not result in death; (3) total cardio-vascular events as a composite of cardio-vascular death, MI, or stroke; (4) stroke as ischemic or haemorrhagic stroke that may or may not have resulted in death; (5) cardio-vascular mortality as death related to CHD or stroke; and (6) all-cause mortality as death related to any cause | None |
| Berger, 2011 [19] | To update a previous meta-analysis that included six trials to test null hypothesis that there is no net benefit relative to risk for aspirin for patients without clinical CVD | Search: MEDLINE, the Cochrane Central Register of Controlled Trials (CCTR), and EMBASE. Inclusion criteria: Aspirin alone was used for the primary prevention of CVD; Comparisons of outcomes were made between aspirin and placebo or open control groups; Data were available on MI, stroke, and cardiovascular deaths. Analysis: Meta-regression. Quality assessment: None | Risk ratio (RR) of aspirin therapy compared with placebo or control on the composite end point, which includes nonfatal MI, nonfatal stroke, or cardiovascular death. All MI, all stroke, all-cause mortality, and cardiovascular mortality. Occurrence of major bleeding | Not clearly distinguished - Major bleeding and haemorrhagic stroke |
| Raju, 2011 [37] | To perform a meta-analysis of all randomised controlled trials on aspirin for the primary prevention of CVD | Search: Medline, EMBASE, CINAHL, Cochrane library, clinicaltrials.gov., references of articles, related items search in PubMed, contacted experts. Inclusion criteria: Randomized controlled trial; adults without a history of symptomatic CVD (>95% of enrolled participants); Compare aspirin (any dose) with placebo or no aspirin treatment for the prevention of CVD; Report at least one of the following outcomes: all-cause mortality, cardiovascular mortality, myocardial infarction, stroke, and bleeding. Analysis: Pooling individual trial data with the DerSimonian-Laird random-effects model. Quality assessment: Cochrane Risk of bias | All-cause mortality, cardiovascular mortality, major cardiovascular events, MI, all-cause stroke, ischemic stroke, haemorrhagic stroke, gastrointestinal bleed, major bleed | Not clearly distinguished - Haemorrhagic stroke, gastrointestinal bleed and major bleeds |
| Raju, 2012 [51] | To critically examine recent meta-analyses comparing aspirin with placebo or no aspirin for the primary prevention of CVD | Search: Medline (2007-2012). Inclusion criteria: Not reported. Analysis: Review of reviews. Quality assessment: Strength of recommendation/level of evidence rating | All-cause and cardiovascular mortality, MI, stroke, major cardiovascular events, bleeding | No new data |
| Selak, 2010 [52] | To model benefit vs harm of aspirin for primary prevention of CVD for age group, gender and risk categories using data from ATT Collaboration meta-analysis | Search: No search reported. Inclusion criteria: Trials on randomised comparison of aspirin versus no aspirin. Analysis: Rates of benefit (avoided vascular events) and harm (additional major extracranial bleeds) for each gender and age group were calculated from data from the six randomised controlled trials included in the Anti-Thrombotic Trialists’ (ATT) Collaboration meta-analysis^41^. Quality assessment: None | Cardiovascular events and serious side effects (extracranial bleeding). Vascular events: MI, stroke (haemorrhagic or other), or death from a vascular cause (coronary heart disease death, stroke death, or other vascular death- including sudden death, death from pulmonary embolism, and death from any haemorrhage) | Not clearly distinguished - as Baigent (2009)^41^ |
| Seshasai, 2012 [38] | To provide an updated synthesis of evidence regarding the wider role of aspirin in primary prevention of CVD and cancer | Search: Pubmed and Cochrane Library until June 2011. Inclusion criteria: Randomized placebo-controlled trials (primary prevention studies) with at least 1000 participants (without previous Coronary Heart Disease [CHD] or stroke), and had at least one-year of follow-up during which CHD and/or CVD outcomes (CHD, stroke, cerebrovascular disease, heart failure, and peripheral arterial disease [PAD]) were recorded as the main end points, and details were provided of bleeding events. Analysis: Study-level meta-analysis. Quality assessment: Delphi scoring system | Total Coronary Heart Disease and total cancer mortality. Subtypes of vascular disease, total CVD events, cause specific death and all-cause mortality. Nontrivial bleeding (fatal bleeding from any site; cerebrovascular or retinal bleeding; bleeding from hollow viscus; bleeding requiring hospitalization and/or transfusion; or study-defined major bleeding regardless of source) | Not clearly distinguished - total bleeds, non-trivial bleeds |
| Wolff, 2009 [11] | To update previous review (2002 USPSTF review) and focuses on new evidence on the benefits and harms of aspirin for the primary prevention of CVD | Search: PubMed and Cochrane central register 2001-2008, Inclusion criteria: Studies that evaluated aspirin versus control for the primary prevention of CVD events in adults. Analysis: No meta-analysis; synthesised qualitatively. Quality assessment: U.S. Preventive Services Task Force (USPSTF) criteria | Not clearly reported; some discussion about bleeds | No new data |
| **RCTs** |  |  |  |  |
| Dorresteijin, 2011 [54] | To identify women who benefit from aspirin 100 mg on alternate days for primary prevention of vascular events by using treatment effect prediction based on individual patient characteristics | Study design: Randomized controlled trial data from the Women’s Health Study | Occurrence of major cardiovascular events (i.e. non-fatal myocardial infarction, non-fatal stroke, or death from cardiovascular causes) | Major and minor bleeds, treatment-induced GI-bleeds/peptic ulcers, haematuria, Epistaxis, and easy bruising |
| Fowkes, 2010 [10] | To determine the effectiveness of aspirin in preventing events in people with a low ABI identified on screening the general population. To determine whether screening the general population for a low ABI could identify a higher-risk group who might derive substantial benefit from aspirin therapy | Study design: A pragmatic intention-to-treat, double-blind, randomized controlled trial | Composite of initial fatal or nonfatal coronary event or stroke or revascularization. All initial vascular events defined as a composite of a primary end point event or angina, intermittent claudication, or transient ischemic attack. All-cause mortality | Major haemorrhage, fatal subarachnoid or subdural haemorrhages, haemorrhagic stroke, fatal and nonfatal subarachnoid/ subdural, fatal and nonfatal gastrointestinal, gastrointestinal ulcer retinal haemorrhage, severe anaemia |
| Nelson, 2008 [53] | To determine the feasibility of performing a large clinical trial of the use of aspirin for the primary prevention of CVD in older participants--the ASPirin in Reducing Events in the Elderly (ASPREE) trial | Study design: Randomised double-blind placebo-controlled pilot trial | The level of response to participation by GPs; the level of response from potential trial participants; the screening-to-randomisation rate to ensure the recruitment target could be achieved; and the retention of participants in the trial after 12 months. Fatal and non-fatal stroke and coronary events. Dementia and clinically significant bleeding (haemorrhagic stroke or gastrointestinal bleeding requiring transfusion or hospitalisation) | Gastrointestinal and intracranial bleeding - Adverse events were determined by patient and investigator report, a search of the medical record held by the practice, and further tracing of data to source documents in specialist and hospital records |

** We only considered adverse events attributable to aspirin. If the outcomes were thought to indicate aspirin benefit but have been called “adverse events” we have written “not clearly distinguished”*
