# Supplementary material for: Aspirin in Primary Prevention of Cardiovascular Disease and Cancer: A Systematic Review of the Balance of Evidence from Reviews of Randomized Trials
Source: PLoS One. 2013 Dec 5;8(12):e81970. doi: 10.1371/journal.pone.0081970 (PMC3855368; doi:10.1371/journal.pone.0081970)
Supplement: Table S9 — Summary characteristics of included systematic reviews investigating aspirin in the primary prevention cancer. (DOCX) [file pone.0081970.s012.docx]

**Table S9. Summary characteristics of included systematic reviews investigating aspirin in the primary prevention cancer**

| **Systematic reviews (First author, year)** | **Aims** | **Methods** | **Outcomes*** | **Adverse events** |
| --- | --- | --- | --- | --- |
| Algra, 2012 [27] | To compare effects of aspirin on risk and outcome of cancer in observational studies versus randomised trials | Search: PubMed (only for case control and cohort studies), trials from Rothwell et al (2011) [28] Inclusion criteria: for RCTs: RCT of aspirin versus no aspirin, mean treatment duration of 4 years or more. Analysis: study-level meta-analysis. Quality assessment: no formal quality assessment | Death, incidence of colorectal cancer, death due to cancer, cancers with distant metastasis | Not reported |
| Mills, 2012 [55] | To determine whether cancer mortality is also reduced in the shorter term | Search: extensive database search. Inclusion criteria: RCTs evaluating low dose, daily aspirin. Analysis: Study level meta-analysis. Quality assessment: quality assessment without validated tool | Non-cardiovascular death and cancer death | Not reported |
| Rothwell, 2010 [13] | To establish the effects of aspirin on incidence and mortality due to colorectal cancer in relation to dose of aspirin and duration of trial | Search: no formal search. Inclusion criteria: RCTs on daily aspirin versus control, minimum of 1000 participants. Median scheduled treatment period of 2.5 years | Death due to colorectal cancer and incidence of colorectal cancer | Not reported |
| Rothwell, 2011 [28] | To determine the effect of aspirin on risk of fatal cancer by analysis of individual patient data for deaths due to cancer during randomised trials of daily aspirin versus control | Search: Trials from ATT collaboration review, PubMed, Embase and Cochrane Database Inclusion criteria: Randomised trials of aspirin (any dose) versus control with a mean duration of trial treatment of at least 4 years. Analysis: IPD meta-analysis. Quality assessment: No formal quality assessment | Total cancer mortality, all-cause mortality, death by site of primary cancer | Not reported |
| Rothwell, 2012 [20] | To establish the effect of aspirin on cancer incidence and the time course of effects on cancer incidence | Search: trials from ATT review, PubMed, Cochrane database and EMBASE. Inclusion criteria: RCTs on daily aspirin, Exclusion of short-term-trials (≤90 days) and trials in the treatment or prevention of secondary cancer or colonic polyps. Analysis: IPD meta-analysis. Quality assessment: no formal quality assessment | Non-vascular death, cancer incidence and cancer death | Major extra-cranial bleeds |
| Rothwell, 2012 [29] | To study metastasis at initial diagnosis and during subsequent follow-up in all participants with a new diagnosis of cancer | Search: refers to Rothwell 2011 and 2012. Inclusion criteria: UK trials of daily aspirin versus control, Exclusion of trials with <10 incident cancers, trials of short-term (≤90 days) treatment and trials in the treatment or prevention of secondary cancer or colonic polyps. Analysis: IPD meta-analysis. Quality assessment: no formal quality assessment | Incidence and mortality due to cancer, cancer metastasis | Not reported |

*primary outcome was generally not reported
