# Supplementary material for: Aspirin in Primary Prevention of Cardiovascular Disease and Cancer: A Systematic Review of the Balance of Evidence from Reviews of Randomized Trials
Source: PLoS One. 2013 Dec 5;8(12):e81970. doi: 10.1371/journal.pone.0081970 (PMC3855368; doi:10.1371/journal.pone.0081970)
Supplement: Table S10 — Summary characteristics of included systematic reviews and RCTs investigating aspirin in the primary prevention cardiovascular events in patients with diabetes. (DOCX) [file pone.0081970.s013.docx]

**Table S10. Summary characteristics of included systematic reviews and RCTs investigating aspirin in the primary prevention cardiovascular events in patients with diabetes**

| **Systematic reviews (First author, year)** | **Aims** | **Methods** | **Outcomes** | **Adverse events** |
| --- | --- | --- | --- | --- |
| Butalia, 2011 [33] | To quantify treatment effects in absolute terms of the risk-benefit trade-off of aspirin therapy in patients with diabetes | Search: Medline, PubMed, Embase, Cochrane library, BIOSIS. Inclusion criteria: RCTs of aspirin versus placebo or vitamins, adults ≥18 years with diabetes without previous historical or clinical evidence of CVD. Analysis: study-level meta-analysis. Quality assessment: JADAD | Primary: MACE (composite of non-fatal MI, non-fatal ischemic stroke, cardiovascular death due to myocardial infarction and ischemic stroke) and all-cause mortality. Secondary: total MI, total stroke, cardiovascular death | Haemorrhage, gastrointestinal bleeding and other gastrointestinal events |
| Calvin, 2009 [34] | To determine whether the effect of aspirin in the primary prevention of cardiovascular events differs between patients with and without diabetes | Search: comprehensive search. Inclusion criteria: RCTs of aspirin versus placebo, patients with diabetes without previous historical evidence of myocardial infarction, Analysis: study level meta-analysis. Quality assessment: quality assessment without validated tool | Ischemic stroke, myocardial infarction and all-cause mortality | Not reported |
| De Berardis, 2009 [35] | To evaluate the benefits and harms of low dose aspirin in people with diabetes and no CVD | Search: Medline, Cochrane central register of controlled trials. Inclusion criteria: RCTs with >500 participants of aspirin versus placebo or no treatment, patients with diabetes mellitus and no CVD. Analysis: study-level meta-analysis. Quality assessment: quality assessment without validated tool | Primary: major cardiovascular event. Secondary: All-cause mortality, death from cardiovascular causes, non-fatal MI and non-fatal stroke | Any bleeding, gastrointestinal bleeding, gastrointestinal symptoms, incidence of cancer |
| Simpson, 2011 [36] | To explore the relationship between aspirin dose and prevention of cardiovascular events | Search: comprehensive search. Inclusion criteria: RCTs, patients with diabetes with or without prior cardiovascular event, aspirin (any dose) versus placebo. Analysis: study-level meta-analysis. Quality Assessment: 27-item checklist | Primary: all-cause mortality. Secondary: cardiovascular-related mortality, MI, stroke | Not reported |
| Stavrakis, 2011 [32] | To evaluate the effect of low-dose aspirin for the primary prevention of cardiovascular event in patients with diabetes mellitus | Search: Medline, EMBASE. Inclusion criteria: RCTs on aspirin versus placebo or no treatment, patients with diabetes and no history of cardiovascular events. Analysis: study-level meta-analysis. Quality assessment: JADAD | Total mortality, cardiovascular mortality (deaths from MI or stroke), major adverse cardiovascular events (death from cardiovascular causes, non-fatal MI, non-fatal stroke),MI (fatal and non-fatal), stroke (fatal and non-fatal) | Major bleeding events including gastrointestinal bleeding |
| Younis, 2010 [31] | To evaluate the benefits of aspirin in people with diabetes mellitus for the primary prevention of CVD | Search: Medline and Cochrane Database. Inclusion criteria: RCTs, diabetic patients, aspirin as a primary prevention of CVD versus placebo or no aspirin. Analysis: study-level meta-analysis. Quality assessment: no formal quality assessment | Major cardiovascular event (composite of cardiovascular death, non-fatal myocardial infarction and stroke), total mortality, myocardial infarction, ischemic stroke | Bleeding |
| Zhang, 2010 [30] | To determine the effect of aspirin therapy in the prevention of cardiovascular events in patients with diabetes | Search: Medline, EMBASE and Cochrane Central Register of controlled trials. Inclusion criteria: RCTs on aspirin versus control, participants with diabetes, at least 12 months follow-up . Analysis: study level meta-analysis. Quality assessment: no formal quality assessment | Major cardiovascular events, all-cause mortality, cardiovascular mortality, MI, and stroke | Major bleeding |
| **RCTs** |  |  |  |  |
| Belch, 2008 [3] | To assess whether aspirin and antioxidant therapy combined or alone, are more effective than placebo in reducing the development of cardiovascular events in patients with diabetes mellitus and asymptomatic peripheral arterial disease | Inclusion criteria: adults aged ≥40 with type 1 or 2 diabetes and an ankle brachial pressure index of 0.99 or less, no symptomatic CVD. Intervention: daily aspirin versus placebo | Primary: death from coronary heart disease or stroke, non-fatal myocardial infarction or stroke, or amputation above ankle or critical limb ischaemia, death from coronary heart disease or stroke | Malignancy, Gastrointestinal bleeding, Gastrointestinal symptoms, Arrhythmia, Allergy including skin rash |
| Ogawa, 2008 [4] | To investigate the efficacy of low-dose aspirin for primary prevention of atherosclerotic events in patients with type 2 diabetes | Inclusion criteria: people with Type 2 diabetes mellitus, age 30-85 years, able to give informed consent. Intervention: daily aspirin versus no aspirin | Primary: any atherosclerotic event (composite of: sudden death, death from coronary, cerebrovascular, and aortic causes, non-fatal acute myocardial infarction, unstable angina, newly developed exertional angina, non-fatal ischemic and haemorrhagic stroke, transient ischemic attack, non-fatal aortic and peripheral vascular disease) | Gastrointestinal events, haemorrhagic events other than haemorrhagic stroke |
