# Supplementary material for: Aspirin in Primary Prevention of Cardiovascular Disease and Cancer: A Systematic Review of the Balance of Evidence from Reviews of Randomized Trials
Source: PLoS One. 2013 Dec 5;8(12):e81970. doi: 10.1371/journal.pone.0081970 (PMC3855368; doi:10.1371/journal.pone.0081970)
Supplement: References S1 — Additional references provided in Tables S8 and S9. (DOCX) [file pone.0081970.s014.docx]

Supporting Information: Additional references provided in Tables S8 and S9

(50) Adelman EE, Lisabeth L, Brown DL (2011) Gender differences in the primary prevention of stroke with aspirin. Women's health 7: 341-352.

(51) Raju NC, Eikelboom JW (2012) The aspirin controversy in primary prevention. Current Opinion in Cardiology 27: 499-507.

(52) Selak V, Elley CR, Wells S, Rodgers A, Sharpe N (2010) Aspirin for primary prevention: yes or no? Journal of Primary Health Care 2: 92-99.

(53) Nelson MR, Reid CM, Ames DA, Beilin LJ, Donnan GA, et al. (2008) Feasibility of conducting a primary prevention trial of low-dose aspirin for major adverse cardiovascular events in older people in Australia: results from the ASPirin in Reducing Events in the Elderly (ASPREE) pilot study. Medical Journal of Australia 189: 105-109.

(54) Dorresteijn JA, Visseren FL, Ridker PM, Paynter NP, Wassink AM, et al. (2011) Aspirin for primary prevention of vascular events in women: individualized prediction of treatment effects. European Heart Journal 32: 2962-2969.

(55) Mills EJ, Wu P, Alberton M, Kanters S, Lanas A, et al. (2012) Low-dose aspirin and cancer mortality: a meta-analysis of randomized trials. American Journal of Medicine 125: 560-567.
